# Integrated Analysis of Differentially Expressed miRNAs and mRNAs in Goat Skin Fibroblast Cells in Response to Orf Virus Infection Reveals That cfa-let-7a Regulates Thrombospondin 1 Expression

**DOI:** 10.3390/v12010118

**Published:** 2020-01-17

**Authors:** Feng Pang, Xinying Wang, Zhen Chen, Zhenxing Zhang, Mengmeng Zhang, Chengqiang Wang, Xiaohong Yang, Qi An, Li Du, Fengyang Wang

**Affiliations:** 1 College of Animal Science and Technology, Hainan University, Hainan Key Lab of Tropical Animal Reproduction & Breeding and Epidemic Disease Research, Haikou 570228, China; 19871122asdf@163.com (F.P.); cz912949981@163.com (Z.C.); zxzhang23@163.com (Z.Z.); mmz2020127@163.com (M.Z.); qiangcw1210@outlook.com (C.W.); wyxiaohong@163.com (X.Y.); I.Angie@outlook.com (Q.A.); kych2008dl@163.com (L.D.); 2 Guizhou Institute of Technology, Guiyang, 550003, China; 20140541@git.edu.cn

**Keywords:** Orf virus, GSF cells, interaction between virus and host cells, mRNAs, miRNAs, cfa-let-7a, THBS1

## Abstract

Orf is a zoonotic disease that has caused huge economic losses globally. Systematical analysis of dysregulated cellular micro RNAs (miRNAs) in response to Orf virus (ORFV) infection has not been reported. In the current study, miRNA sequencing and RNA sequencing (RNA-seq) were performed in goat skin fibroblast (GSF) cells at 0, 18, and 30 h post infection (h.p.i). We identified 140 and 221 differentially expressed (DE) miRNAs at 18 and 30 h.p.i, respectively. We also identified 729 and 3961 DE genes (DEGs) at 18 and 30 h.p.i, respectively. GO enrichment analysis indicates enrichment of apoptotic regulation, defense response to virus, immune response, and inflammatory response at both time points. DE miRNAs and DEGs with reverse expression were used to construct miRNA-gene networks. Seven DE miRNAs and seven DEGs related to “negative regulation of viral genome replication” were identified. These were validated by RT-qPCR. Cfa-let-7a, a significantly upregulated miRNA, was found to repress Thrombospondin 1 (THBS1) mRNA and protein expression by directly targeting the THBS1 3′ untranslated region. THBS1 has been reported to induce apoptosis; therefore, the cfa-let-7a-THBS1 axis may play an important role in cellular apoptosis during ORFV infection. This study provides new insights into ORFV and host cell interaction mechanisms.

## 1. Introduction

Orf, also known as contagious ecthyma, is a zoonotic disease that has led to great economic losses in livestock production globally [1]. The disease mainly affects goats and sheep, but also affects other ruminants and mammals such as musk ox, steenbok, reindeer, dog, and cat [1,2]. Moreover, people can become infected following contact with infected animals [3]. ORFV, a member of the genus *Parapoxvirus*, is the causative agent of Orf. ORFV has a double-stranded DNA genome of approximately 130–140 kb, encoding 132 genes [4]. The relatively conserved central regions of the ORFV genome are responsible for morphogenesis and viral replication while the highly variable terminal regions are responsible for virus virulence [5].

Various primary cell cultures and cell lines were used to isolate ORFV. Initially, primary lamb testis and primary lamb kidney cells were commonly used for ORFV isolation [6]. Primary fetal lamb muscle cells [7], ovine fetal turbinate cells [8], and goat skin fibroblast cells [9] have also been widely used for ORFV isolation. Madin–Darby bovine and Madin–Darby ovine kidney are the most commonly used cell lines for ORFV isolation and propagation [10]. To date, the majority of research on ORFV has focused on the functions of its virulence genes. Virulence factors that have been identified include an IL-10-like gene [5], chemokine binding protein (CBP, [11]), vascular endothelial growth factor (VEGF, [12]), apoptosis inhibitor ORF125 [13], interferon (IFN) resistance gene [14], and inhibitors of NF-κB such as ORF002, ORF024, and ORF121 [15,16,17].

Micro RNAs (miRNAs) are small non-coding RNAs of approximately 22 nucleotides in length that are found in animals, plants, and some viruses [18,19,20,21]. They play crucial roles in multiple biological processes including cancer, apoptosis, and immune response [18,22,23,24]. Cellular miRNAs can regulate viral replication by targeting the expression of cellular or viral genes [25,26]. MiRNAs usually function by targeting mRNA in the 3′ untranslated region (3′UTR) and suppressing protein synthesis [27]. Many algorithms have been developed for miRNA target prediction. The miRanda algorithm is mainly based on binding energy of miRNA-3′UTR, evolutionary conservation of target sites, and position within the 3′UTR [28]. The TargetScan algorithm ranks projected targets by either the predicted efficacy of targeting (context+ scores) or the probability of conserved targeting (PCT) [27].

**Figure 1:**
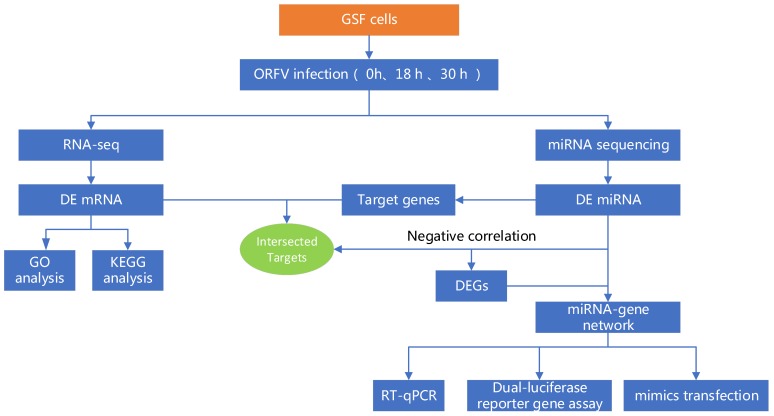
Flow chart of the present study.

The role of miRNAs in ORFV infection and the mechanisms by which ORFV and host cells interact remain largely unknown. Therefore, in the present study, we performed miRNA sequencing and RNA-seq at three different infection times (0, 18, 30 h). Differentially expressed miRNAs and genes were analyzed at 18 and 30 h.p.i to identify potential ORFV responsive miRNA-gene regulatory networks existing in GSF cells. Figure 1 provides a summary flow chart of the present work. The current study provides new insights into ORFV-host interaction mechanisms.

## 2. Materials and Methods 

### 2.1. Cell Culture and Viral Infection

GSF or HEK293T cells were purchased from the Cell Bank of the Chinese Academy of Science (Kunming or Shanghai, China) and maintained in Dulbecco’s modified Eagle medium (DMEM; Invitrogen, Carlsbad, CA, USA), supplemented with 10% fetal bovine serum (Invitrogen). For ORFV infection, GSF cells (90% confluent) were infected with ORFV JS strain (TCID50 = 10^6.2^/mL) at a multiplicity of infection (MOI) of 1. After 1 h (h) of incubation, the supernatant was removed and cells were cultured for another 18 or 30 h.

### 2.2. RNA Extraction

Total RNA from uninfected GSF cells, GSF cells at 18 h.p.i and 30 h.p.i (triplicates of each group) were isolated using an Ambion mirVana™ miRNA isolation kit (Thermo Fisher Scientific, Waltham, MA, USA). The integrity and concentration of total RNA were analyzed using an Agilent 2100 bioanalyzer (Agilent Technologies, Santa Clara, CA, USA) and a NanoDrop™ 2000 (Thermo Fisher Scientific, Lafayette, CO, USA), respectively. Total RNA with RNA integrity number (RIN) >7 was used for high-throughput sequencing.

### 2.3. miRNA Sequencing and RNA-seq

As previously described, equivalent total RNA from 18 and 30 h.p.i GSF samples was used for miRNA sequencing and RNA-seq on an Illumina HiSeq 2500 or 4000 platform respectively (LC Sciences, Hangzhou, China) [29]. For miRNA analysis, raw reads from each sample were subjected to ACGT101-miR, an in-house program developed by LC Sciences, to acquire clean reads. Subsequently, unique sequences with length in 18–26 nucleotide were mapped to *Capra hircus* precursors in miRBase 21.0 to identify known and novel miRNAs. L/R ± n meant that the detected miRNA sequence was n base more/less than known miRNA in the left/right side. Read counts to tags per million counts (TPM) was used to normalize the expression levels of miRNAs. For RNA-seq analysis, raw data was filtered by Cutadapt to acquire clean reads [30]. Then, clean reads were aligned to the *Capra hircus* reference genome (Accession number: GCF_001704415.1) using the HISAT package [31]. The mapped reads of each sample were assembled using StringTie, which was then used to perform expression level for mRNAs by calculating fragments per kilobase of exon per million reads mapped (FPKM) [32].The cutoffs for DE miRNAs and DEGs were fold change ≥2 or fold change ≤0.5, and *p* ≤ 0.05. The raw data have been deposited in the Gene Expression Omnibus (Accession numbers: GSE141162 and GSE141163).

### 2.4. GO and KEGG Enrichment Analyses

To understand the biological functions and pathways of the enriched DEGs, we performed GO and KEGG pathway enrichment analyses as we previously described [29]. GO terms or pathways with *p* ≤ 0.05—calculated by hypergeometric test, relative to the whole genome—were significantly enriched. 

### 2.5. Target Gene Prediction and miRNA-Gene Network Construction

MiRanda 3.3a and TargetScan 7.0 algorithms were used to predict miRNA targets in the *Capra hircus* genome (GCF_001704415.1). Target genes with a context score percentile of less than 50 in the TargetScan algorithm and with max free energy values > –10 in MiRanda were removed. DE miRNAs and DEGs with inverse expression were used to build miRNA-gene networks using Cytoscape 3.6.0 software [29].

### 2.6. RT-qPCR Validation of DEGs and DE miRNAs

For DEG validation, total RNA from each sample was used to prepare cDNA using a HiScript III first strand cDNA synthesis kit (Vazyme, Nanjing, China). Then, qPCR was performed using the ChamQ universal SYBR qPCR master mix (Vazyme) on an ABI 7500 real-time PCR system (Applied Biosystems, Foster City, CA, USA). The miRNA first strand cDNA synthesis kit (Vazyme) and the miRNA universal SYBR qPCR master mix (Vazyme) were used for miRNA validation per manufacturer’s protocols. The relative expression levels of genes or miRNAs (normalized to goat glyceraldehyde-3-phosphate dehydrogenase (GAPDH) or U6 snRNA, respectively) were calculated by the 2^−ΔΔCt^ method. All experiments were performed in triplicate.

### 2.7. Cell Transfection

GSF cells were seeded at a density of 1 × 10^5^ cells/mL in a 24-well plate. Upon reaching approximately 60% confluence, cells were transfected with 100 nM cfa-let-7a_R+2 mimic or negative NC 22 control mimic (RioBio, Guangzhou, China) using lipofectamine RNAiMAX reagent (Thermo Fisher Scientific, Lafayette, Colorado, USA) per the manufacturer’s protocol. After 48 h, cells were rinsed three times with PBS and lysed with an RNeasy animal RNA isolation kit (Beyotime, Shanghai, China).

### 2.8. Western Blot 

GSF cells were transfected with 100 nM cfa-let-7a_R+2 mimic or NC 22 control mimic. After 48 h, total protein was extracted using IP lysis buffer (Beyotime) containing 1mM phenylmethylsulfonyl fluoride (PMSF). Protein concentration was determined using the Pierce BCA protein assay kit (Thermo Fisher Scientific, Lafayette, Colorado, USA). Approximately 50 μg total protein was used for Western blot (WB). A mouse monoclonal anti-thrombospondin 1 (THBS1) antibody (1:1000, Santa Cruz Biotechnology, Santa Cruz, CA, USA) and a rabbit anti-mouse IgG-horseradish peroxidase (HRP) secondary antibody (1:5000, Cell Signaling Technology, Danvers, MA, USA) were used to detect THBS1 protein. A goat polyclonal antibody against GAPDH (1:2000, Santa Cruz Biotechnology) and a rabbit anti-goat IgG antibody, HRP conjugate (1:5000, Boster Biotechnology company, Wuhan, China) were used to detect the GAPDH internal control. 

### 2.9. Plasmid Construction 

The partial 3′ UTR of THBS1 containing a cfa-let-7a_R+2 binding site was amplified from cDNA of GSF cells by PCR and subcloned into the PmeI-XhoI site of the pmirGLO dual-luciferase miRNA target expression vector (Promega, Madison, WI, USA). A mutated THBS1 3′UTR reporter (pmirGLO-THBS1 mut-3′UTR) was generated by mutating the seed region (UACCUC→AUGGAG) of the cfa-let-7a_R+2 by overlap extension PCR. The recombinant plasmids, pmirGLO-THBS1 wt-3′UTR and pmirGLO-THBS1 mut-3′UTR, were extracted using an endofree plasmid midi kit (Aidlab, Beijing, China) and sequenced by Tianyi Huiyuan Biotech (Guangzhou, China).

### 2.10. Dual Luciferase Reporter Assay

HEK293T cells were seeded in a 24-well plate (1×10^5^ cells per well) one day prior to transfection. When cells reached approximately 60% confluence, pmirGLO-THBS1 wt-3′UTR and pmirGLO-THBS1 mut-3′UTR (100 ng) plasmids were co-transfected with 100 nM negative NC 22 control mimic or cfa-let-7a_R+2 mimic (RiboBio, Guangzhou, China) using the lipofectamine RNAiMAX reagent (Thermo Fisher Scientific) per the manufacturer’s protocol. The relative luciferase activity (Firefly/ Renilla) was measured 48 h after transfection using the dual-luciferase reporter assay (Vazyme) on a modulus single tube multimode reader (Tuner Biosystems, USA). Six replicates of each co-transfection were performed.

### 2.11. Statistical Analysis

Two-tailed Students’ T-test was used to evaluate the significance of the dual luciferase reporter assay and WB using GraphPad Prism 5 software. *p* ≤ 0.05 was considered statistically significant. Data are shown as mean ± SD from three independent experiments.

## 3. Results

### 3.1. Differentially Expressed miRNAs From Intergroup Comparisons

**Figure 2:**
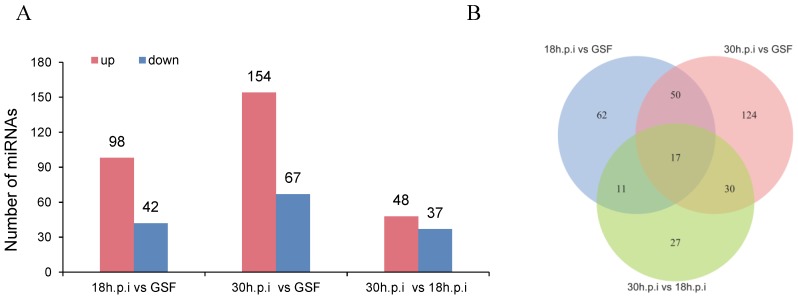
Differentially expressed miRNAs in 18h.p.i vs. GSF, 30h.p.i vs. GSF, and 30h.p.i vs. 18h.p.i. (**A**) Bar chart of DE miRNAs in 18h.p.i vs. GSF, 30h.p.i vs. GSF, and 30h.p.i vs. 18h.p.i. (**B**) Venn diagram of DE miRNAs in 18h.p.i vs. GSF, 30h.p.i vs. GSF, and 30h.p.i vs. 18h.p.i.

In a previous study, we investigated changes in circular RNAs, miRNAs, and mRNAs at the early stage of ORFV infection [9]. In the current study, we focused on DE miRNAs and DEGs during the late stage of ORFV infection—18 and 30 h.p.i. Filtered raw reads yielded a total of 1465, 1438, and 1415 miRNAs in the GSF, 18, and 30 h.p.i groups, respectively (Figure S1). Among these, 1151 miRNAs were common in all three groups. The length distribution of miRNAs in all libraries was similar, with the majority being 22 nucleotides long. At 18 and 30 h.p.i, 98 and 154 miRNAs were upregulated, respectively, while 42 and 67 miRNAs were downregulated, respectively. Compared with the 18 h.p.i group, 48 miRNAs were upregulated and 37 miRNAs were downregulated at 30 h.p.i (Figure 2A, Tables S1–S3). Venn diagrams identified 67 DE miRNAs shared by the 18 h.p.i vs. GSF and 30 h.p.i vs. GSF comparisons and 17 DE miRNAs shared by the 18 h.p.i vs. GSF, 30 h.p.i vs. GSF, and 30 h.p.i vs. 18 h.p.i comparisons (Figure 2B). 

### 3.2. KEGG Enrichment Analyses of DE miRNAs From Intergroup Comparisons

**Figure 3:**
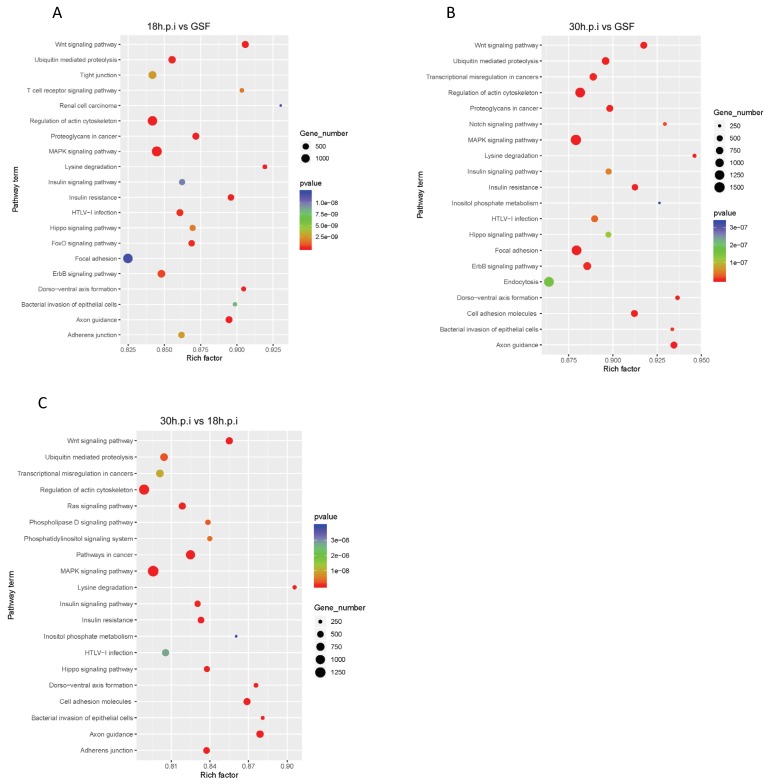
KEGG enrichment analyses of DE miRNAs in 18h.p.i vs. GSF, 30h.p.i vs. GSF and 30h.p.i vs. 18h.p.i. (**A**) Top 20 significantly enriched KEGG pathways of DE miRNAs in 18h.p.i vs. GSF. (**B**) Top 20 significantly enriched KEGG pathways of DE miRNAs in 30h.p.i vs. GSF. (**C**) Top 20 significantly enriched KEGG pathways of DE miRNAs in 30h.p.i vs. 18h.p.i

To explore potential functions of miRNAs, DE miRNAs from the three comparison groups were evaluated using the TargetScan and miRanda algorithms. Predicted target genes from the analyses were then subjected to KEGG enrichment analyses. There were 121, 113, and 128 significantly enriched KEGG pathways identified in the 18 h.p.i vs. GSF, 30 h.p.i vs. GSF, and 30 h.p.i vs. 18 h.p.i comparisons, respectively (Tables S4–S6). The wingless-related integration site (Wnt), mitogen activated protein kinase (MAPK), T cell receptor, chemokine, tumor necrosis factor (TNF), and nuclear factor-kappa B (NF-κB) signaling pathways were shared by all three comparison groups. The top 20 significantly enriched pathways (*p* ≤ 0.05) in the 18 h.p.i vs. GSF, 30 h.p.i vs. GSF, and 30 h.p.i vs. 18 h.p.i comparisons are presented in Figure 3.

### 3.3. DEGs Identified by Intergroup Comparisons

RNA-seq of each sample yielded 44,324,190 to 57,183,142 raw reads (Table 1). After filtering low quality reads, a mean of 51,202,435, 50,443,699, and 43,420,612 clean reads were obtained from GSF, 18 h.p.i, and 30 h.p.i group, respectively. Ratios of clean reads were all above 96% in each sample. More than 93% of clean reads in GSF samples were mapped to the goat reference genome. In comparison, approximately 53% and 38% of clean reads were aligned to the goat genome in 18 and 30 h.p.i samples, respectively. The mapping percentage of clean reads to goat genome in ORFV-infected samples reduced sharply in a time-dependent manner.

**Figure 4:**
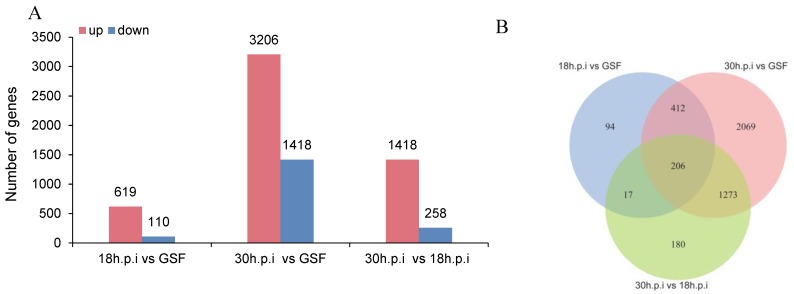
DEGs in 18h.p.i vs. GSF, 30h.p.i vs. GSF and 30h.p.i vs. 18h.p.i. (**A**) Bar chart of DEGs in 18h.p.i vs. GSF, 30h.p.i vs. GSF, and 30h.p.i vs. 18h.p.i. (**B**) Venn diagram of DEGs in 18h.p.i vs. GSF, 30h.p.i vs. GSF, and 30h.p.i vs. 18h.p.i.

In the 18 h.p.i vs. GSF comparison, 619 upregulated and 110 downregulated genes were identified. In the 30 h.p.i vs. GSF comparison, 3206 upregulated and 755 downregulated genes were identified. In the 30 h.p.i vs. 18 h.p.i comparison, 1418 upregulated and 258 downregulated genes were identified (Figure 4A, Tables S7–S9); Venn diagram analysis showed that 618 genes were differentially expressed in both the 18 h.p.i vs. GSF and 30 h.p.i vs. GSF comparisons and 206 genes were shared between the three intergroup comparisons (Figure 4B). 

### 3.4. GO Enrichment Analyses of DEGs From Intergroup Comparisons

**Figure 5:**
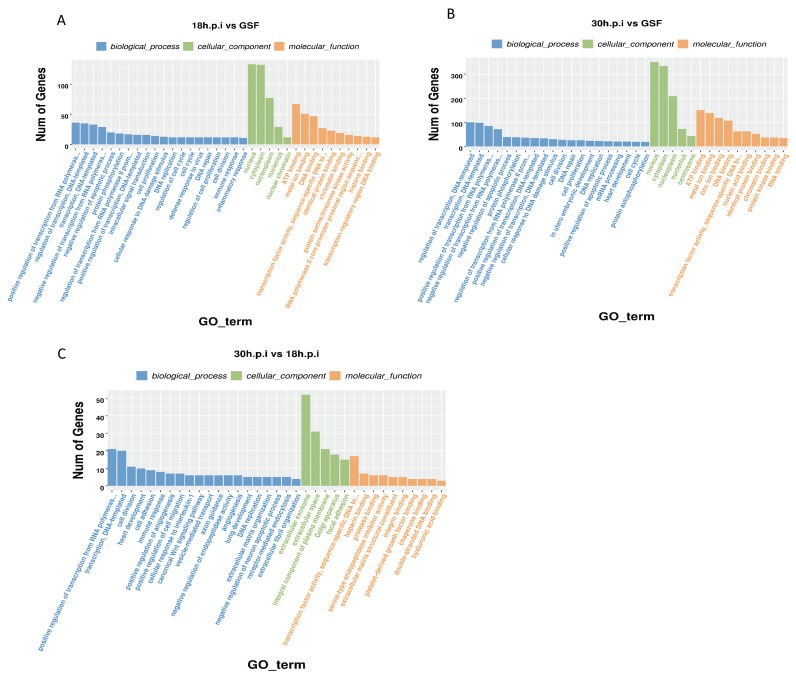
GO enrichment analyses of DEGs in 18h.p.i vs. GSF, 30h.p.i vs. GSF and 30h.p.i vs. 18h.p.i. (**A**) GO enrichment analysis of DEGs in 18h.p.i vs. GSF. (**B**) GO enrichment analysis of DEGs in 30h.p.i vs. GSF. (**C**) GO enrichment analysis of DEGs in 30h.p.i vs. 18h.p.i.

**Figure 6:**
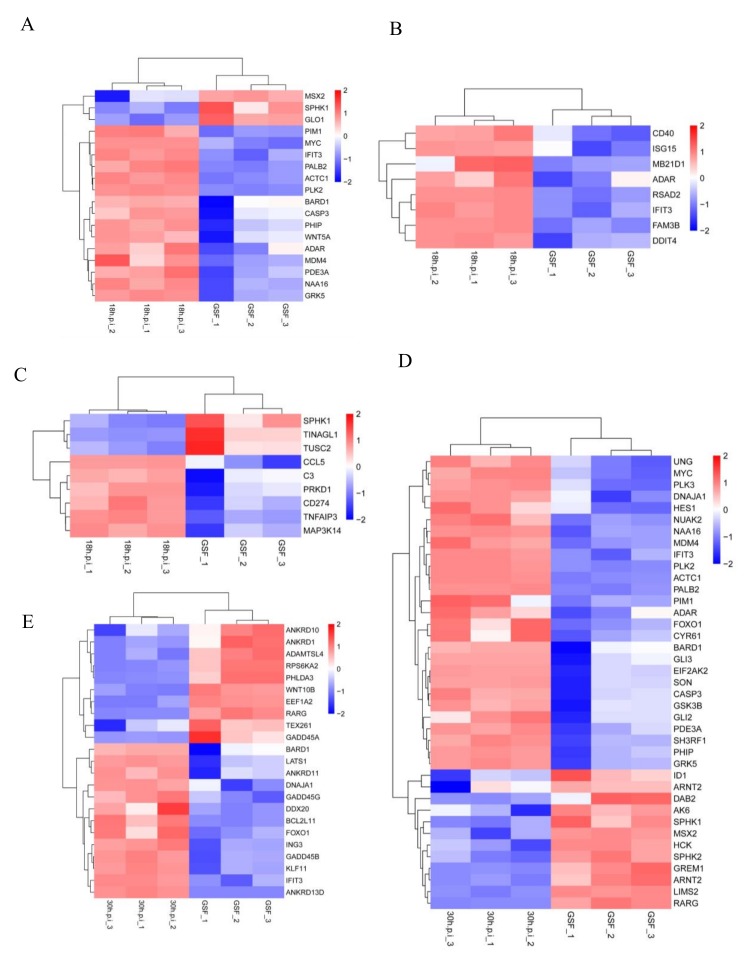
The clustered heatmaps of cellular immune response genes in the 18h.p.i vs. GSF comparison and positive and negative regulation of apoptosis genes identified in the 30h.p.i vs. GSF comparison. (**A**) Heatmap of DEGs enriched in negative regulation of apoptotic process in the 18h.p.i vs. GSF comparison. (**B**) Heatmap of DEGs enriched in defense response to virus in the 18h.p.i vs. GSF comparison. (**C**) Heatmap of DEGs enriched in immune and inflammatory response in the 18h.p.i vs. GSF comparison. (**D**) Heatmap of DEGs enriched in negative regulation of apoptotic process in the 30h.p.i vs. GSF comparison. (**E**) Heatmap of DEGs enriched in positive regulation of apoptotic process in the 30h.p.i vs. GSF comparison.

DEGs that were enriched in the 18 h.p.i. vs. GSF comparison were mainly associated with negative regulation of apoptotic process, cell cycle, defense response to virus, immune response, and inflammatory response (Figure 5A). DEGs that were enriched in the 30 h.p.i. vs. GSF comparison were mainly associated with cell cycle, positive regulation of apoptosis, and negative regulation of apoptosis (Figure 5B). DEGs that were enriched in the 30 h.p.i. vs. 18 h.p.i. comparison were mainly associated with negative regulation of apoptosis, immune response, and canonical Wnt signaling pathway (Figure 5C). The clustered heatmaps of cellular immune response genes in the 18 h.p.i vs. GSF comparison and positive and negative regulation of apoptosis genes identified in the 30 h.p.i vs. GSF comparison are shown in Figure 6.

### 3.5. KEGG Enrichment Analyses of DEGs From Intergroup Comparisons

**Figure 7:**
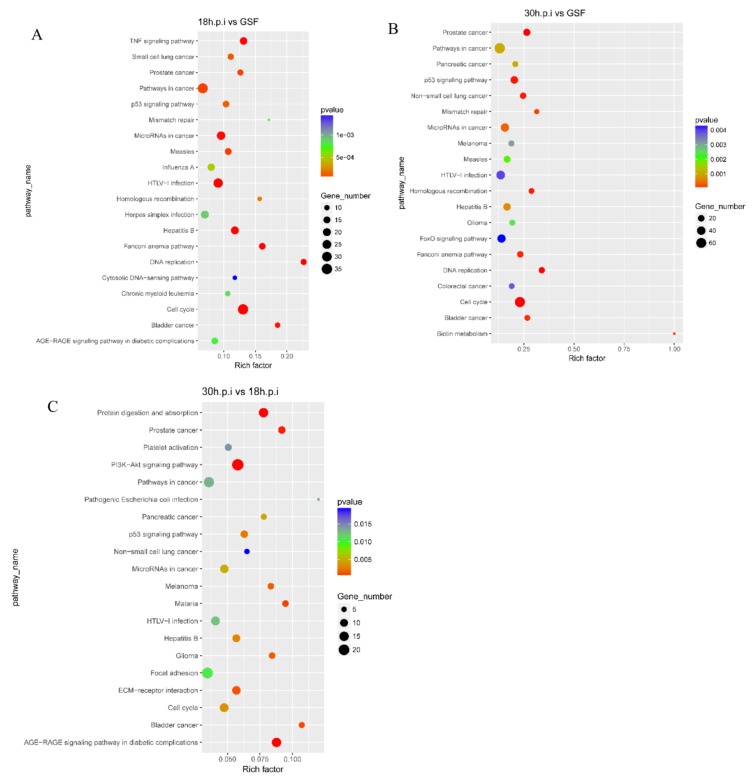
KEGG enrichment analyses of DEGs in 18h.p.i vs. GSF, 30h.p.i vs. GSF and 30h.p.i vs. 18h.p.i. (**A**). KEGG enrichment analysis of DEGs in 18h.p.i vs. GSF. (**B**) KEGG enrichment analysis of DEGs in 30h.p.i vs. GSF. (**C**) KEGG enrichment analysis of DEGs in 30h.p.i vs. 18h.p.i.

A total of 42, 37, and 34 pathways were significantly enriched in the 18 h.p.i vs. GSF, 30 h.p.i vs. GSF, and 30 h.p.i vs. 18 h.p.i comparisons, respectively. The top 20 significantly enriched pathways identified in the three comparisons are presented in Figure 5. In the 18 h.p.i vs. GSF comparison, cell cycle, TNF, p53, Toll-like receptor, NF-κB, and chemokine signaling pathways were significantly enriched (Figure 7A). In the 30 h.p.i vs. GSF comparison, cell cycle, p53, TNF, phosphoinositide 3-kinase -protein kinase B (PI3K-Akt), and apoptosis pathways were significantly enriched (Figure 7B). In the 30 h.p.i vs. 18 h.p.i comparison, PI3K-Akt, p53, cell cycle, and cytokine-cytokine receptor interaction pathways were significantly enriched (Figure 7C).

### 3.6. RT-qPCR Validation of DE miRNAs and DEGs

**Figure 8:**
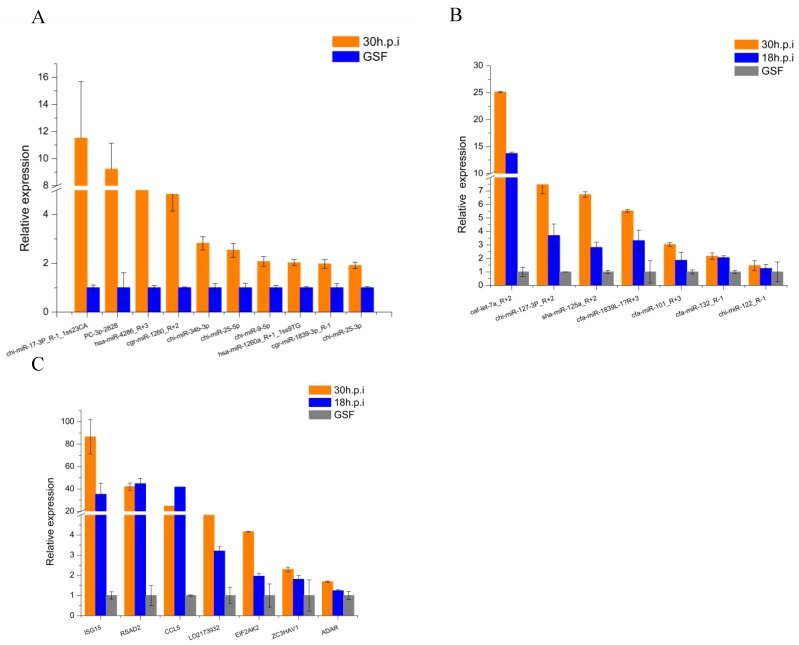
RT-qPCR validation of DE miRNAs and DEGs. (**A**) RT-qPCR validation of DE miRNAs in 30h.p.i vs. GSF. (**B**) RT-qPCR validation of DE miRNAs in both 18h.p.i vs. GSF and 30h.p.i vs. GSF. (**C**) RT-qPCR validation of DEGs.

To assess the reliability of high-throughput sequencing, 10 DE miRNAs identified from the 30 h.p.i vs. GSF comparison and 7 DE miRNAs identified in both the 18 h.p.i vs. GSF and 30 h.p.i vs. GSF comparisons (TPM > 50) were selected for RT-qPCR validation. Results indicate that relative expression of 10 miRNAs in the 30 h.p.i vs. GSF comparison were consistent with results from miRNA sequencing (Figure 8A). Among these, chi-miR-17-3P_R-1_1ss23C, PC-3p-282, hsa-miR-4286_R+3, and cgr-miR-1260_R+2 were all upregulated more than 4-fold. Seven miRNAs showed increased expression in both the 18 h.p.i vs. GSF and 30 h.p.i vs. GSF comparisons: cfa-let-7a_R+2 (hsa-let-7a_R+2), chi-miR-127-3P_R+2, sha-miR-125a_R+2, cfa-miR-1839_L-1 R+3, cfa-miR-101_R+3, cfa-miR-132_R-1, and chi-miR-122_R-1 (Figure 8B). Cfa-let-7a_R+2 was upregulated more than 20-fold. 

DEGs enriched for the “negative regulation of viral genome replication” GO term (Table 2) were selected for RT-qPCR validation. Seven genes were identified as upregulated in response to ORFV infection. Interferon-stimulated gene 15 (ISG15), radical S-adenosyl methionine domain containing 2 (RSAD2), and C-C motif chemokine ligand 5 (CCL5) were all upregulated more than 20-fold in both the 18 h.p.i vs. GSF and 30 h.p.i vs. GSF comparisons (Figure 8C). The primers used for RT-qPCR of selected miRNAs and genes are listed in Tables S10–S12.

### 3.7. cfa-let-7a_R+2 Target Prediction and Validation 

**Figure 9:**
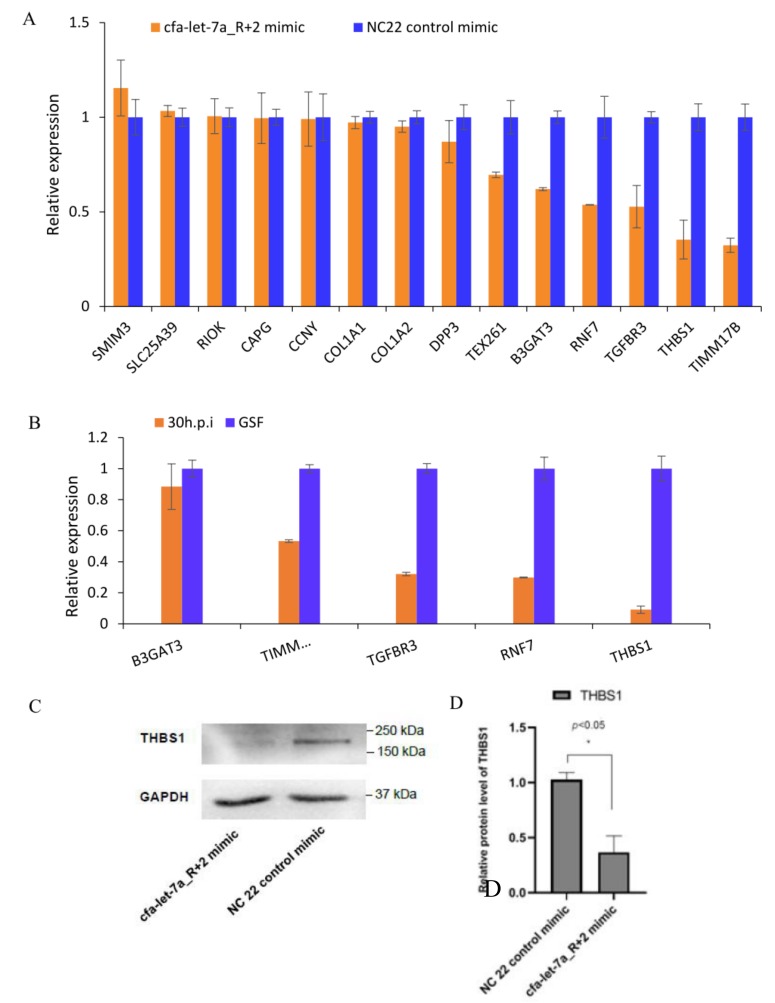
cfa-let-7a_R+2 target prediction and validation. (**A**) RT-qPCR validation of cfa-let-7a_R+2 targets after Table S7. a_R+2 targets in RNA-seq. (**B**) RT-qPCR validation of cfa-let-7a_R+2 targets in RNA-seq. (**C**) WB detection of THBS1 protein expression after transfecting miRNA mimics. (**D**) Gray analysis of THBS1 protein expression. *P* < 0.05 means significant difference.

Among the validated DE miRNAs, cfa-let-7a_R+2 was the most upregulated, therefore it was chosen for further analysis. Target gene prediction identified 55 potential targets for this miRNA. Fourteen of the identified genes had relatively high expression with FPKM >10. To verify the 14 predicted targets, cfa-let-7a_R+2 mimic or NC 22 control mimic was transfected into GSF cells. Following 48 h of incubation, RT-qPCR was conducted to detect the mRNA expression levels of the 14 targets. Results indicate that cfa-let-7a_R+2 mimic significantly reduced the mRNA expression level of beta-1,3-glucuronyltransferase 3 (*B3GAT3),* ring finger protein 7 (*RNF7*), transforming growth factor beta receptor 3 (*TGFBR3*), thrombospondin 1 (*THBS1*), and translocase of inner mitochondrial membrane 17B (*TIMM17B*) (Figure 9A). Significant downregulation of RNF7, TGFBR3, THBS1, and TIMM17B was verified by RT-qPCR (Figure 9B).

Overall, THBS1 was downregulated much more than other four cfa-let-7a targets based on the above results. Furthermore, previous studies reported that THBS1 was related to apoptosis [33,34] which was an important immune mechanism used by cells against viral infection. Therefore, we first selected THBS1 for Western blot analysis. THBS1 protein expression levels were detected after 48 h following cfa-let-7a_R+2 mimic or NC 22 control mimic transfection. Results indicate that cfa-let-7a_R+2 mimic significantly reduced THBS1 protein expression compared with the control mimic (Figure 9C,D).

### 3.8. cfa-let-7a_R+2 Directly Targets THBS1 3′ UTR

**Figure 10:**
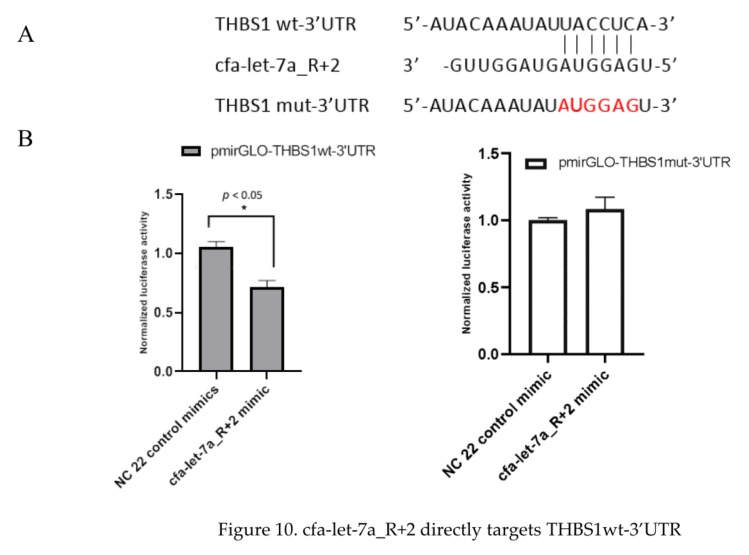
cfa-let-7a_R+2 directly targets THBS1wt-3’UTR. (**A**) The wild and mutant binding sites between THBS1-3’UTR and cfa-let-7a_R+2. The red representing the mutant binding sites. (**B**) cfa-let-7a_R+2 inhibiting the luciferase activity of pmirGLO-THBS1wt-3’UTR but not that of pmirGLO-THBS1 mut-3’UTR.

The mechanism by which cfa-let-7a_R+2 represses THBS1 was investigated. Dual-luciferase reporter vectors containing the wild-type cfa-let-7a target sequences of THBS1 3′UTR (pmirGLO-THBS1 wt-3′UTR) and mutant cfa-let-7a target sequences (pmirGLO-THBS1 mut-3′UTR) were constructed and luciferase reporter assays were performed. Constructs were co-transfected with cfa-let-7a_R+2 mimic or NC 22 control mimic into HEK293T cells. Co-transfection of cfa-let-7a_R+2 mimic with the wild type reporter led to 40% decrease in luciferase activity; whereas co-transfection with the mutant reporter resulted in no change in luciferase activity (Figure 10), indicating that cfa-let-7a_R+2 bound to the THBS1 3′UTR region. Based on these findings, we postulate that cfa-let-7a_R+2 directly targets THBS1 3′UTR to suppress expression of THBS1 mRNA and protein. 

## 4. Discussion

High-throughput sequencing is a powerful tool for investigating virus-host interaction. So far, this tool has been used limitedly in researching ORFV-host interactions. Therefore, in the current study, we performed miRNA sequencing and RNA-seq at 0, 18, and 30 h post ORFV infection. We identified 140 and 221 DE miRNAs at 18 and 30 h.p.i., respectively. We also identified 729 and 3961 DEGs at 18 and 30 h.p.i, respectively. GO enrichment analysis revealed that the main categories of DEG were: positive or negative regulation of apoptotic process, defense response to virus, immune response, and inflammatory response. KEGG enrichment analysis revealed association of the DEGs with TNF, p53, Toll-like receptor, NF-κB, chemokine, PI3K-Akt, and apoptosis signaling pathways.

7 DEGs related to “negative regulation of viral genome replication” were identified by RT-qPCR. Expression of ISG15 and RSAD2 displayed a sharp time-dependent increase following ORFV infection. ISG15 is an IFN-stimulated gene that encodes a ubiquitin-like protein [35,36]. ISG15 has been reported to be a broad-spectrum antiviral protein against both DNA and RNA viruses, including herpes simplex type-1, influenza A and B, HIV-1, hepatitis B and E, Ebola virus, and respiratory syncytial virus [37,38,39,40,41,42,43]. RSAD2 also known as cig5 and Viperin, is a highly conserved protein expressed in most cell types. It has been reported to be induced by double-stranded DNA, RNA, lipopolysaccharide, IFN, and a number of viruses [44]. RSAD2 has demonstrated antiviral activity against a broad range of viruses, including human cytomegalovirus, hepatitis C virus, dengue virus, influenza virus, West Nile virus, and chikungunya virus [45,46,47,48,49,50]. Whether ISG15 and RASD2 have an anti-ORFV function remains to be further studied. 

Ten miRNAs DE at 30 h.p.i and 7 miRNAs DE at both 18 and 30 h.p.i were validated. One of the significantly upregulated miRNA was identified as cfa-let-7a_R+2 (hsa-let-7a_R+2). Hsa-let-7a has been reported to be downregulated in various types of human cancer including nasopharyngeal carcinoma, papillary thyroid carcinoma, lung cancer, and cervical cancer [51,52,53,54]. One study reported that hsa-let-7a inhibits migration, invasion, and tumor growth by targeting AKT2 in papillary thyroid carcinoma [51]. Another reports that hsa-let-7a inhibits proliferation and induces apoptosis by targeting EZH2 in nasopharyngeal carcinoma cells [52]. Moreover, hsa-let-7a has been shown to elevate p21WAF1 levels by targeting NIRF and suppressing the growth of A549 lung cancer cells [53].

In the current study, cfa-let 7a was identified to suppress THBS-1 mRNA and protein expression by directly targeting THBS1 3′UTR. THBS-1 is a multifunctional extra-cellular matrix glycoprotein secreted by multiple types of cells including endothelial cells, monocytes, and fibroblasts [33,55]. THBS-1 is an endogenous inhibitor of angiogenesis [56]. A previous study reported that THBS-1 inhibits angiogenesis and induces endothelial cell apoptosis [33]. Zhu et al. demonstrated that miR-222 inhibits apoptosis in porcine follicular granulosa cells by suppressing the expression of THBS1 [34]. Based on previous studies and the current results, we postulate that cfa-let-7a suppresses cellular apoptosis by targeting THBS1, which would be beneficial for ORFV replication in GSF cells. This assumption needs to be further confirmed in the future study. 

## Figures and Tables

**Table 1:** Statistics of raw and clean reads from each sample.

Sample	Raw Reads	Clean Reads	Mapped Reads	Q20	Q30
GSF_1	57,183,142	55,444,222	51,842,931(93.50%)	99.30	96.27
GSF_2	48,776,996	48,162,078	45,704,129(94.90%)	99.27	95.70
GSF_3	50,682,932	50,001,006	46,891,314(93.78%)	99.40	95.78
18 h.p.i_1	54,753,068	54,206,124	28,379,819(52.36%)	99.21	95.43
18 h.p.i_2	52,356,386	51,858,906	27,326,358(52.69%)	99.21	95.38
18 h.p.i_3	45,717,786	45,266,068	24,495,939(54.12%)	98.80	94.50
30 h.p.i_1	42,852,548	42,351,874	16,204,107(38.26%)	99.00	94.89
30 h.p.i_2	44,324,190	43,961,096	16,296,813(37.07%)	98.85	94.52
30 h.p.i_3	44,336,038	43,948,868	16,994,445(38.67%)	98.74	94.21

**Table 2:** DEGs (fold change ≥2 or fold change ≤0.5, and *p* ≤ 0.05 ) enriched in “negative regulation of viral genome replication” GO term. The number in the table represents the FPKM of genes in GSF, 18 h.p.i, and 30 h.p.i groups.

Gene	GSF_1	GSF _2	GSF _3	18 h.p.i_1	18 h.p.i _2	18 h.p.i_3	30 h.p.i_1	30 h.p.i_2	30 h.p.i_3
*EIF2AK2*	1.63	10.39	9.56	22.17	21.29	22.44	42.28	41.02	44.33
*RSAD2*	0.10	0.05	0.13	11.90	11.65	12.40	12.80	10.66	11.40
*ISG15*	2.90	0.14	0.34	16.07	17.61	15.28	38.51	30.52	33.22
*CCL5*	1.75	0.24	0.05	17.42	16.22	17.91	9.32	8.77	7.14
*ADAR*	5.53	6.92	12.24	14.08	16.98	20.23	17.53	13.94	22.05
*ZC3HAV1*	4.72	14.67	14.01	14.66	13.39	30.56	41.63	40.32	41.62
*LOC102173932*	1.15	2.89	2.76	8.51	8.13	8.31	24.96	24.20	25.12

## Supplementary Materials

The following are available online at https://www.mdpi.com/1999-4915/12/1/118/s1, Figure S1: Venn diagram of miRNAs detected in GSF samples at 0, 18, and 30 h.p.i. Figure S2: Predicted target genes of cfa-let-7a_R+2. Table S1: Differentially expressed miRNAs in 18 h.p.i vs. GSF. Table S2: Differentially expressed miRNAs in 30 h.p.i vs. GSF. Table S3: Differentially expressed miRNAs in 30 h.p.i vs. 18 h.p.i. Table S4: KEGG pathway enrichment analysis of DE miRNAs in 18 h.p.i vs. GSF. Table S5. KEGG pathway enrichment analysis of DE miRNAs in 30 h.p.i vs. GSF. Table S6. KEGG pathway enrichment analysis of DE miRNAs in 30 h.p.i vs. 18 h.p.i. Table S7. Differentially expressed genes in 18 h.p.i vs. GSF. Table S8. Differentially expressed genes in 30 h.p.i vs. GSF. Table S9. Differentially expressed genes in 30 h.p.i vs. 18 h.p.i. Table S10. Primers of selected common DE miRNAs in 30 h.p.i vs. GSF and 18 h.p.i vs. GSF. Table S11. Primers of selected DE miRNAs in 30 h.p.i vs. GSF. Table S12. Primers of DEGs enriched in “negative regulation of viral genome replication”.
