# Supplementary material for: Integrated Analysis of Differentially Expressed miRNAs and mRNAs in Goat Skin Fibroblast Cells in Response to Orf Virus Infection Reveals That cfa-let-7a Regulates Thrombospondin 1 Expression
Source: Viruses. 2020 Jan 17;12(1):118. doi: 10.3390/v12010118 (PMC7019303; doi:10.3390/v12010118)
Supplement: Supplementary file 1 [file viruses-12-00118-s001.zip › Supplementary materials/Table S10.pdf]

Table S10. Primers of selected common DE miRNAs in 30h.p.i vs GSF and 18h.p.i vs GSF.

Primer name	Sequence (5' to 3')
chi-miR-122 R-1 loop	GTCGTATCCAGTGCAGGGTCCGAGGTATTCGCACTGGATACGACAAACAC
chi-miR-122 R-1 -F	CGCGTGGAGTGTGACAATG
cfa-miR-132-R-1 loop	GTCGTATCCAGTGCAGGGTCCGAGGTATTCGCACTGGATACGACCGACCA
cfa-miR-132-R-1 -F	GCGCGTAACAGTCTACAGCCA
cfa-miR-1839L-1R+3 loop	GTCGTATCCAGTGCAGGGTCCGAGGTATTCGCACTGGATACGACTCACAA
cfa-miR-1839 L-1R+3 -F	CGCGAGGTAGATAGAACAGGTC
cfa-let-7a_R+2 loop	GTCGTATCCAGTGCAGGGTCCGAGGTATTCGCACTGGATACGACTTAACT
cfa-let-7a_R+2 -F	GCGCGTGAGGTAGTAGGTTGTAT
cfa-miR-101_R+3 loop	GTCGTATCCAGTGCAGGGTCCGAGGTATTCGCACTGGATACGACTCTTCA
cfa-miR-101_R+3 -F	CGCGCGTACAGTACTGTGATAAC
sha-miR-125a_R+2 loop	GTCGTATCCAGTGCAGGGTCCGAGGTATTCGCACTGGATACGACTCTCAC
sha-miR-125a_R+2 -F	GCGTCCCTGAGACCCTAACTT
chi-miR-127-3p_R+2 loop	GTCGTATCCAGTGCAGGGTCCGAGGTATTCGCACTGGATACGACAGCCAA
chi-miR-127-3p_R+2 -F	CGTCGGATCCGTCTGAGC
Universal Reverse Primer	AGTGCAGGGTCCGAGGTATT
U6-F	CGCTTCGGCAGCACATATACTA
U6-R	CGCTTCACGAATTTGCGTGTCA
