# Supplementary material for: Integrated Analysis of Differentially Expressed miRNAs and mRNAs in Goat Skin Fibroblast Cells in Response to Orf Virus Infection Reveals That cfa-let-7a Regulates Thrombospondin 1 Expression
Source: Viruses. 2020 Jan 17;12(1):118. doi: 10.3390/v12010118 (PMC7019303; doi:10.3390/v12010118)
Supplement: Supplementary file 1 [file viruses-12-00118-s001.zip › Supplementary materials/Table S11.pdf]

Table S11. Primers of selected DE miRNAs in 30h.p.i vs GSF.

Primer name	Sequence (5' to 3')
cgr-miR-1839-3p_R-1 loop	GTCGTATCCAGTGCAGGGTCCGAGGTATTCGCACTGGATACGACTGTTGG
cgr-miR-1839-3p_R-1 -F	CGCGCGAGACCTACTTATCTA
hsa-miR-4286_R+3 loop	GTCGTATCCAGTGCAGGGTCCGAGGTATTCGCACTGGATACGACTTTGGT
hsa-miR-4286_R+3 -F	GCGACCCCACTCCTGGT
cgr-miR-1260_R+2 loop	GTCGTATCCAGTGCAGGGTCCGAGGTATTCGCACTGGATACGACTATGGT
cgr-miR-1260_R+2 -F	CGATCCCACCGCTGCC
hsa-miR-1260a_R+1_1ss9TG loop	GTCGTATCCAGTGCAGGGTCCGAGGTATTCGCACTGGATACGACTTGGTG
hsa-miR-1260a_R+1_1ss9TG -F	GCGATCCCACCGCTGC
PC-3p-2828_1480 loop	GTCGTATCCAGTGCAGGGTCCGAGGTATTCGCACTGGATACGACTCAAAA
PC-3p-2828_1480 -F	CGCGAAAAAACCGAGTGAACT
chi-miR-25-3p loop	GTCGTATCCAGTGCAGGGTCCGAGGTATTCGCACTGGATACGACTCAGAC
chi-miR-25-3p -F	GCGCATTGCACTTGTCTCG
chi-miR-25-5p loop	GTCGTATCCAGTGCAGGGTCCGAGGTATTCGCACTGGATACGACAGCAAT
chi-miR-25-5p -F	AGGCGGAGACTTGGGCA
chi-miR-9-5p loop	GTCGTATCCAGTGCAGGGTCCGAGGTATTCGCACTGGATACGACTCATAC
chi-miR-9-5p -F	GCGCGTCTTTGGTTATCTAGCT
chi-miR-34b-3p loop	GTCGTATCCAGTGCAGGGTCCGAGGTATTCGCACTGGATACGACGATGGC
chi-miR-34b-3p -F	GCGCGAATCACTAGTTCCACT
chi-miR-17-3p_R-1_1ss23CA loop	GTCGTATCCAGTGCAGGGTCCGAGGTATTCGCACTGGATACGACTCTACA
chi-miR-17-3p_R-1_1ss23CA -F	GCGACTGCAGTGAAGGCACT
Universal Reverse Primer	AGTGCAGGGTCCGAGGTATT
U6-F	CGCTTCGGCAGCACATATACTA
U6-R	CGCTTCACGAATTTGCGTGTCA
