# Supplementary material for: Integrated Analysis of Differentially Expressed miRNAs and mRNAs in Goat Skin Fibroblast Cells in Response to Orf Virus Infection Reveals That cfa-let-7a Regulates Thrombospondin 1 Expression
Source: Viruses. 2020 Jan 17;12(1):118. doi: 10.3390/v12010118 (PMC7019303; doi:10.3390/v12010118)
Supplement: Supplementary file 1 [file viruses-12-00118-s001.zip › Supplementary materials/Table S12.pdf]

Table S12. Primers of DEGs enriched in "negative regulation of viral genome replication".

Primer name	Sequence (5'to3')
EIF2AK2-F	GGCTGCCAAACTGGCTTATG
EIF2AK3-R	CCCACACGCGGTATTGAAAC
RSAD2-F	CCCACCAGCGTCAATTACCA
RSAD2-R	CACGAAGGACGTTTTGGCTG
ISG15-F	CGGACCAATTCTGGCTGTCT
ISG15-R	CCCCGCAGACGTAGATTCAT
CCL5-F	TCCATGGCAGCAGTTGTCTTT
CCL5-R	TTGATGTACTCTCGCACCCA
ZC3HAV1-F	CCACACCTTCATCCGTCACA
ZC3HAV1-R	CTCAGCTCGTAGTTGCGTGA
LOC102173932-F	GGCCTGCATGTGTTGACAAG
LOC102173932-R	TGGATTGCCCAAGACTGGAC
ADAR-F	TACCAAGCGAAAGTTGGGGG
ADAR-R	GAGAGGGAGCTCTGTGAAACC
GAPDH-F	AGCCGTAACTTCTGTGCTGT
GAPDH-R	TTCCCGTTCTCTGCCTTGAC
