# Supplementary figures and images for: Integrated Analysis of Differentially Expressed miRNAs and mRNAs in Goat Skin Fibroblast Cells in Response to Orf Virus Infection Reveals That cfa-let-7a Regulates Thrombospondin 1 Expression
Source: Viruses. 2020 Jan 17;12(1):118. doi: 10.3390/v12010118 (PMC7019303; doi:10.3390/v12010118)

Figure S1. Venn diagram of miRNAs detected in GSF samples at 0h.p.i, 18h.p.i and 30h.p.i.

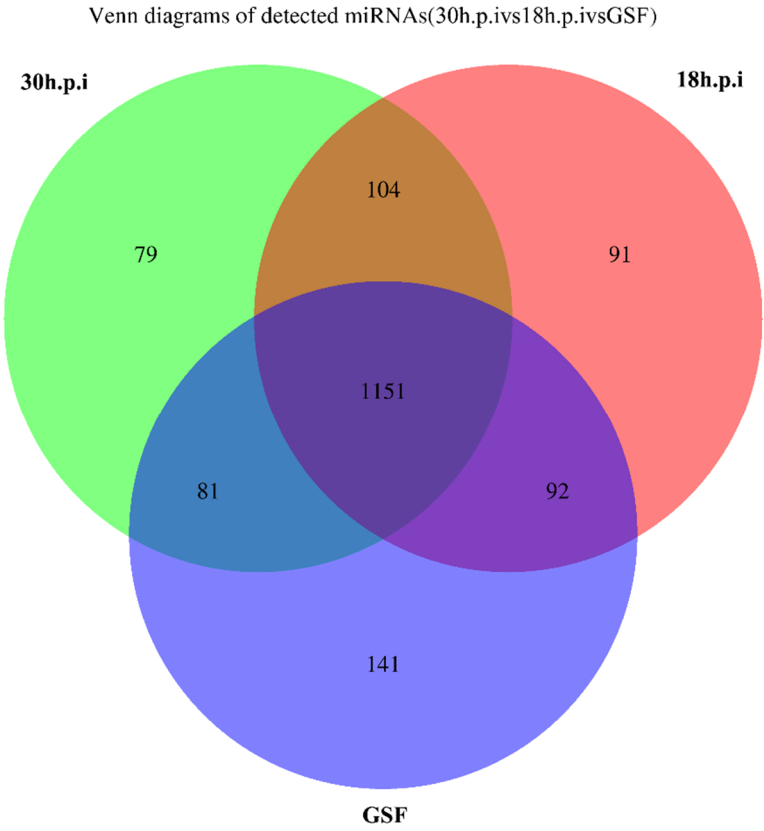

Supplement: Supplementary file 1 [file viruses-12-00118-s001.zip › Supplementary materials/Figure S1.pdf]

Figure S2. Predicted target genes of cfa-let-7a\_R+2.

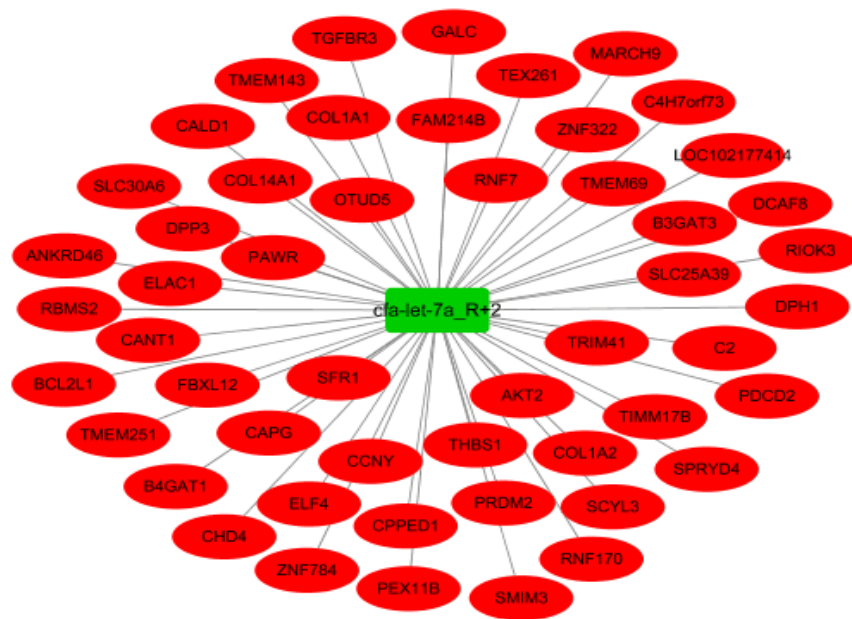

Supplement: Supplementary file 1 [file viruses-12-00118-s001.zip › Supplementary materials/Figure S2.pdf]
